# Transfemoral prosthetic simulators versus amputees: ground reaction forces and spatio-temporal parameters in gait

**DOI:** 10.1098/rsos.231854

**Published:** 2024-03-27

**Authors:** Toshiki Kobayashi, Abu Jor, Yufan He, Mingyu Hu, Mark W. P. Koh, Genki Hisano, Takeshi Hara, Hiroaki Hobara

**Affiliations:** ^1^ Department of Biomedical Engineering, Faculty of Engineering, The Hong Kong Polytechnic University, Hong Kong, People's Republic of China; ^2^ Department of Leather Engineering, Faculty of Mechanical Engineering, Khulna University of Engineering & Technology, Khulna, Bangladesh; ^3^ Faculty of Advanced Engineering, Tokyo University of Science, Tokyo, Japan; ^4^ Japan Society for the Promotion of Science (JSPS), Tokyo, Japan

**Keywords:** ambulation, ground reaction force, kinetics, speed, symmetry, walk

## Abstract

This study aimed to compare the ground reaction forces (GRFs) and spatio-temporal parameters as well as their asymmetry ratios in gait between individuals wearing a transfemoral prosthetic simulator (TFSim) and individuals with unilateral transfemoral amputation (TFAmp) across a range of walking speeds (2.0–5.5 km h^−1^). The study recruited 10 non-disabled individuals using TFSim and 10 individuals with unilateral TFAmp using a transfemoral prosthesis. Data were collected using an instrumented treadmill with built-in force plates, and subsequently, the GRFs and spatio-temporal parameters, as well as their asymmetry ratios, were analysed. When comparing the TFSim and TFAmp groups, no significant differences were found among the gait parameters and asymmetry ratios of all tested metrics except the vertical GRFs. The TFSim may not realistically reproduce the vertical GRFs during the weight acceptance and push-off phases. The structural and functional variations in prosthetic limbs and components between the TFSim and TFAmp groups may be primary contributors to the difference in the vertical GRFs. These results suggest that TFSim might be able to emulate the gait of individuals with TFAmp regarding the majority of spatio-temporal and GRF parameters. However, the vertical GRFs of TFSim should be interpreted with caution.

## 1. Introduction

Transfemoral amputation (TFAmp), whether owing to dysvascularity, trauma or other reasons, is a type of major amputation and has a great and lifelong impact on mobility and quality of life. The worldwide incidence of major lower extremity amputation ranged from 3.6 to 68.4 per 100 000 individuals from 1989 to 2010 [1], and it is projected to double in the USA between 2005 and 2050 [2]. The mobility of individuals with amputation is closely associated with the level of amputation and other factors. Approximately 30% of lower-limb amputations occur at the transfemoral level and 50% at the transtibial level [3]. Using a prosthesis or assistive device can effectively improve mobility. Therefore, in rehabilitation, it is important to better understand the mobility issues of individuals with amputation to enhance the design of prostheses and potentially fully restore their ambulation abilities and functional performance [4].

Clinicians and engineers have been developing not only prostheses for individuals with amputation but also prosthetic simulators for non-disabled individuals. The prosthetic simulators are expected to assist in understanding the gait of individuals with amputation and in facilitating the research and development of prosthetic components, particularly in early-stage prototyping [5,6]. The transfemoral prosthetic simulator (TFSim, also referred to as a bypass device/prosthesis [7]) is a prosthetic device that is fitted on one of the lower limbs of a non-disabled individual to temporarily simulate the gait of a person with unilateral TFAmp (electronic supplementary material, video S1). There are two types of TFSims that have been reported in the literature. Lemaire *et al*. developed a V-shaped ischial-support socket TFSim which is fabricated according to the lower-limb dimensions of each individual using a computer-aided design and manufacturing system [8]. The other type is an L-shaped socket TFSim. This simulator is composed of a socket for accommodating the lower limb with the knee joint flexed at about 100°, with the body mass supported by the anterior surface of the lower limb [9,10]. For either TFSim, a prosthetic knee joint is attached to the socket beneath the anatomical knee joint, and a prosthetic foot is attached distally using a pylon with an adaptor.

A TFSim serves two main purposes. The first purpose is to use it as a tool in the research and development of prosthetic components [7,9]. For example, TFSims have been used in research to evaluate design parameters in transfemoral prostheses, such as the effects of recognition error on volitional control as well as safe and confident use of prostheses [11], knee flexion strategy [10], ankle stiffness [6] and durability of prosthetic components [12]. In addition, TFSims have been used in the development of transfemoral prosthetic components [13–16] and their myoelectric control mechanisms [17,18]. Clinicians and researchers frequently measure gait parameters and their symmetry to evaluate the effectiveness of these components [19]. However, it should be noted that a TFSim might not be able to realistically replicate the gait of an individual with amputation. The discrepancy might compromise the research outcomes or the performance of the designed prosthetic components.

The second purpose of a TFSim is to allow non-disabled individuals to experience some of the challenges during walking of a person with a unilateral TFAmp which serves as a hands-on tool in school and clinical education. It is useful and helpful for students and clinicians to deepen their understanding of the gait of individuals who are using a unilateral transfemoral prosthesis [8,20]. It is therefore essential for TFSims to replicate the gait exhibited by individuals with unilateral TFAmp. Although TFSims are commonly used in educational, clinical, research and developmental settings, there is a lack of studies that compare the gait characteristics of individuals wearing a TFSim and individuals with unilateral TFAmp using a prosthesis. Individuals with unilateral TFAmp often exhibit gait abnormalities characterized by asymmetric gait patterns resulting from compensatory mechanisms. For example, individuals with unilateral TFAmp using a prosthesis walked slower (about 30% reduced speed) when compared with non-disabled individuals and demonstrated asymmetrical gait parameters (e.g. kinematic and kinetic) between the intact and the amputated sides [21]. The asymmetries were also reflected in stride length, step time and ground reaction forces (GRFs) [22,23]. Significant kinematic asymmetries at the knee joint during the stance phase and the ankle joint during the swing phase were reported [24]. In addition, gait asymmetries with respect to GRF were generally found to be greater at higher walking speeds [25]. Besides, the length of the residual limb affects the gait of individuals with unilateral TFAmp. It was observed that those with shorter residual limbs exhibited increased trunk and pelvic excursion [26]. Therefore, a TFSim needs to realistically replicate the gait pattern of an individual with unilateral TFAmp to be used in clinical and research settings.

A previous study evaluated the kinematic and kinetic parameters of individuals with unilateral TFAmp, using literature data, and compared these with five first-time TFSim users. This comparison revealed similarities in ankle joint angular velocities, moments and powers, alongside knee moments and powers [8]. However, it remains unclear whether TFSim can accurately replicate the gait patterns and the asymmetrical differences observed between the intact and amputated limbs. Variations in structural symmetry, such as the differences in the location of the effective knee joint, the whole-body centre of mass and the limb/socket interface, in TFSim when compared with individuals with TFAmp, may lead to changes in gait patterns [27,28]. A direct comparison of gait between the two groups could produce scientific evidence and confirm the effectiveness of the TFSim in replicating biomechanical and spatio-temporal parameters in individuals with unilateral TFAmp. This could provide more insight into the development or prototyping of prosthetic components.

The first aim of this study was to compare spatio-temporal parameters and GRFs (mediolateral, anteroposterior and vertical components) of gait and their asymmetry ratios between individuals wearing a TFSim and individuals with unilateral TFAmp using a prosthesis while walking on an instrumented treadmill across a range of speeds. The second aim of this study was to investigate the effects of gradual changes in gait speed on these parameters when comparing the two groups. The use of TFSim offers the advantage of comparing various prosthetic devices, including prosthetic feet, knees and alignment arrangements, even during the initial stage of development. However, it is unclear whether non-disabled individuals walking with TFSim exhibit consistency in gait compared with individuals with unilateral TFAmp. Therefore, we proposed the following two hypotheses: our first hypothesis (H1) was that there would be significant differences in the spatio-temporal parameters, GRFs and asymmetry ratios between individuals wearing a TFSim and individuals with unilateral TFAmp using prostheses across the range of walking speeds. The second hypothesis (H2) was that an increment in gait speed would have significantly different effects on these parameters when comparing the two groups.

## 2. Material and methods

### 2.1. Participants

The inclusion criteria for non-disabled participants included (i) being aged 18 or older, (ii) having used TFSim for a minimum of 6 months, (iii) engaging in regular TFSim walking training frequently in working places, and (iv) demonstrating the ability to walk on a treadmill across various speeds. For individuals with unilateral TFAmp, the criteria included (i) being aged 18 or older, (ii) having functional levels of K3 or K4, (iii) having no neuromusculoskeletal complications except the amputation itself, and (iv) demonstrating the ability to walk on a treadmill at various speeds. Before conducting the experiment, the purpose of this study and the risks associated with measurement were fully explained to all participants, and their consent was obtained for participation in the study. The Institutional Review Board (Environmental and Safety Headquarters, Safety Management Division, National Institute of Advanced Industrial Science and Technology) ethically approved the study, and the experiments were conducted according to the Declaration of Helsinki (1983).

### 2.2. Experimental procedures

**Figure 1.**
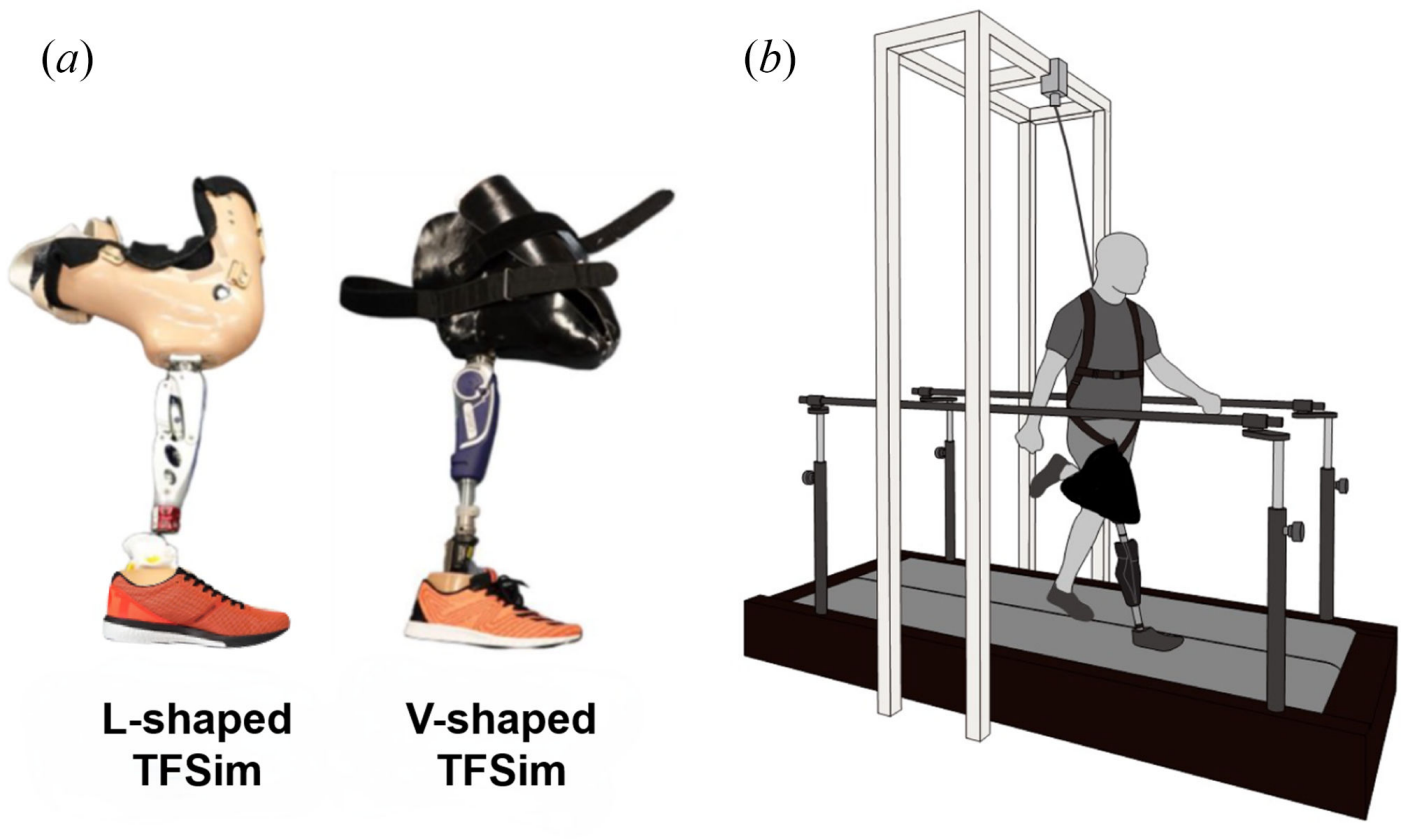
(*a*) TFSim used in the present study. Based on the prosthetic socket design, they are L-shaped and V-shaped TFSim. (*b)* Schematic representation of the experiment with TFSim (V-shaped TFSim). Participants walked on a split-belt force-instrumented treadmill at eight speeds (2.0–5.5 km h^−1^ with an increment of 0.5 km h^−1^) for 30 s per speed. Two force plates were embedded in the split-belt treadmill. A safety harness and two handrails were equipped to prevent falling.

An instrumented treadmill with built-in force plates (FTMH-1244WA; Tech Gihan, Kyoto, Japan) was used to measure GRFs and spatio-temporal parameters (figure 1). The two built-in force plates (TF-40120-CL and TF-40120-CR; Tech Gihan, Kyoto, Japan) measure forces in three directions (mediolateral, Fx; anteroposterior, Fy; vertical, Fz). The treadmill is also equipped with a safety harness device to prevent falls during data collection. Each individual wore their own TFSim or transfemoral prosthesis during data collection. The TFSims were custom-made and aligned individually by a certified prosthetist. For the group wearing a TFSim (figure 1), nine individuals used the V-type, while one individual used the L-type (‘TFSim 6’ in table 1). Before the experiment, the individuals practised walking on the treadmill for 7 min. This was done as previous studies suggested that accurate data acquisition for gait analysis requires at least 5 min of practice with the split-belt treadmill [29]. Through this walking practice, it was confirmed that the individuals became familiar with walking on the treadmill and could walk in the expected speed range. The participants were asked to make sure to keep one foot on each belt of the split-belt treadmill. The range of walking speeds in the experiment was set from 2.0 to 5.5 km h^−1^ with increments of 0.5 km h^−1^. The experiment was conducted across eight different speed conditions for all participants. The data were collected for 30 s at each speed after the speed of the treadmill became stable. During the test, sufficient breaks based on the needs of each participant were provided to eliminate the effects of fatigue. All individuals wore the equipped safety harness to prevent falls during data collection on the instrumented treadmill. Movies of each participant walking at 3.5 km h^−1^ on the treadmill can be found in the electronic supplementary material, video S2, for TFSim and electronic supplementary material, video S3, for TFAmp.

**Table 1.** Demographic data of the participants.

participant (TFSim)	gender	age (years)	height (m)	mass (kg)	BMI	TFSim experience (years)	residual limb length	prosthetic knee unit	prosthetic feet	prosthetic socket
1	male	30	1.67	64.3	23.1	5.5	—	3R95	Flex-Foot Assure	V-type
2	male	28	1.77	75.2	24	6	—	3R95	Flex-Foot Assure	V-type
3	female	26	1.64	60.9	22.6	0.8	—	3R95	Flex-Foot Assure	V-type
4	male	38	1.61	86.6	33.4	5	—	Swan100	J-Foot	V-type
5	female	26	1.65	62.5	23	3	—	Dolphin	J-Foot	V-type
6	female	28	1.62	69.4	26.4	8	—	3R60	Flex-Foot Assure	L-type
7	male	29	1.82	69.3	20.9	3.2	—	3R60	J-Foot	V-type
8	male	28	1.74	76.3	25.2	5	—	3R106	Triton	V-type
9	male	35	1.77	76.4	24.4	5.5	—	3R80	Triton	V-type
10	male	40	1.77	103.4	33	5	—	Total knee	Variflex	V-type
mean	—	30.8	1.71	74.4	25.6	4.7	—	—	—	—
s.d.	—	5	0.08	12.8	4.3	2	—	—	—	—

participant (TFAmp)	gender	age (years)	height (m)	mass (kg)	BMI	time since amputation (years)	residual limb length	prosthetic knee unit	prosthetic feet	cause of amputation
1	male	23	1.68	58.3	20.7	20	short	3R80	Variflex	cancer
2	female	21	1.49	47.5	21.4	10	short	3R106	Total concept	sarcoma
3	female	20	1.56	56.4	23.2	5.7	middle	Total knee	Variflex	trauma
4	male	27	1.75	71	23.2	6.2	middle	3R80	Triton	trauma
5	female	27	1.54	46	19.4	1	middle	3R60	RS2000	trauma
6	male	34	1.61	61.4	23.7	21	middle	3R95	Variflex	sarcoma
7	male	29	1.65	70.1	25.7	7.7	long	3R80	Triton	trauma
8	male	42	1.75	111.1	36.3	24.5	long	3R80	Dyna Trek	trauma
9	male	42	1.7	75.4	26.1	32	short	3R80	Highlander	trauma
10	male	34	1.7	75.4	26.1	18	long	3R80	Variflex	trauma
mean	—	29.9	1.64	67.3	24.6	14.6	—	—	—	—
s.d.	—	8	0.09	18.7	4.7	9.9	—	—	—	—

*Notes:* BMI, body mass index. TFSim experience (years) indicates their years of experience using a TFSim at their workplaces. Definitions of residual limb length are as follows: ‘long’ is longer than two-thirds of the intact limb, but not knee disarticulation; ‘middle’ is less than two-thirds but longer than one-third of the intact limb; ‘short’ is less than one-third of the intact limb.

BMI, body mass index; TFAmp, transfemoral amputation; TFSim, transfemoral prosthetic simulator.

### 2.3. Data collections and analysis

**Figure 2.**
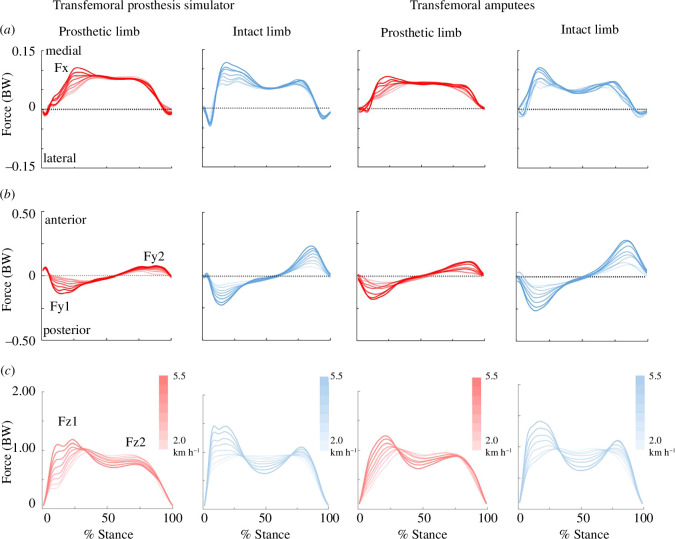
Observed variables derived from GRFs: mediolateral (*a*), anteroposterior (*b*) and vertical (*c*) components for the limbs of both the TFSim group and the group with TFAmp under eight walking speeds. Red (prosthetic limb) and blue (intact limb) curves represent the average GRF values of the TFSim and TFAmp groups, respectively. The lightest and darkest colours represent the slowest (2.0 km h^−1^) and highest walking speeds (5.5 km h^−1^), respectively. As the speed increases, colour darkens. In each plot, the *X*-axis is the normalized stance from 0% to 100%, and the *Y*-axis is the force (normalized with respect to body weight (BW)).

The GRFs (mediolateral, anteroposterior and vertical) at each walking speed were measured at a sampling frequency of 1000 Hz. The acquired data were filtered using a fourth-order Butterworth low-pass filter with a cut-off frequency of 20 Hz [30]. From the vertical GRFs data, the maximum number of consecutive steps on the left and right belts was analysed. In addition, a threshold of 40 N was used for the vertical components of the GRF in each belt to determine the beginning (heel-strike) and ending (toe-off) of the stance [25,31]. The variability of GRFs against time (0–100% stance) for intact and prosthetic limbs of both TFSim and TFAmp groups is shown in figure 2. The pairwise variability in GRFs between the TFSim and TFAmp groups based on knee joints can be found in electronic supplementary material, figure S1. Subsequently, peak values of GRFs were obtained for further analysis. The first peaks of the mediolateral, anteroposterior and vertical GRFs were defined as Fx, Fy1 and Fz1, respectively, based on the force plate coordinate system (figure 2). The second peaks of the anteroposterior and vertical GRFs were defined as Fy2 and Fz2, respectively. Posterior GRFy indicates braking, while anterior GRFy indicates propulsion during stance. The first peak of vertical GRFs indicates weight acceptance in early stance, while the second peak of vertical GRFs indicates push-off in late stance. When there was a double peak in Fz1, the higher peak was selected for further analysis. All GRF data were normalized by body weight with a TFSim/prosthesis. Spatio-temporal parameters, including step length (m), cadence (steps s^−1^), stance time (s), swing time (s) and double limb stance (DLS) time (s), were also determined using the 40 N threshold values. The DLS of the intact limb was defined as the intact limb leading, while the DLS of the prosthetic limb was defined as the prosthetic limb leading [32]. The gait asymmetry was analysed using the asymmetry ratio as a reliable method reported in previous studies, where the ratio of each gait parameter was calculated by dividing the data of the prosthetic limb by the intact limb for both the TFSim and TFAmp groups [33,34]. Therefore, an asymmetry ratio greater or smaller than 1 indicates gait asymmetry.

### 2.4. Statistical analysis

For each gait parameter and asymmetry ratio, the Shapiro–Wilk test was used to check data normality. For gait parameters and asymmetry ratios with a normal distribution, a two-way mixed ANOVA (between-groups: TFSim group and TFAmp group at each speed, within-group: current speed and previous speed, interaction: combined effect of speed and group) was conducted followed by pairwise comparisons (between two groups to address the first hypothesis: H1, between current and previous speeds to address the second hypothesis: H2) with Bonferroni correction. The partial η^2^ was calculated as the effect size (ES). For gait parameters and asymmetry ratios without a normal distribution, the Mann–Whitney *U* test was conducted for the comparison of the TFSim group and TFAmp group at each speed to address the first hypothesis: H1, while the Friedman test followed by the Wilcoxon signed-rank test as a *post hoc* test were conducted to compare current and previous speeds to address the second hypothesis: H2. The critical α was set at 0.05 in all statistical tests used in this study (IBM SPSS Statistics v. 28, IBM, Armonk, NY, USA).

## 3. Results

### 3.1. Participants

Ten non-disabled individuals (three females and seven males, age: 29.9 ± 8.0 years, body mass: 67.3 ± 18.7 kg) who were familiar with walking with a TFSim participated in the TFSim group. Ten individuals (three females and seven males, age: 30.8 ± 5.0 years, body mass: 74.4 ± 12.8 kg) with a unilateral TFAmp accustomed to walking with a transfemoral prosthesis who had functional levels of either K3 or K4 participated in the TFAmp group. The detailed demographic information of the participants can be found in table 1. The non-disabled individuals were clinicians or educators who used TFSim regularly at their workplaces.

### 3.2. Spatio-temporal parameters and asymmetry

#### 3.2.1. Stance time

**Figure 3.**
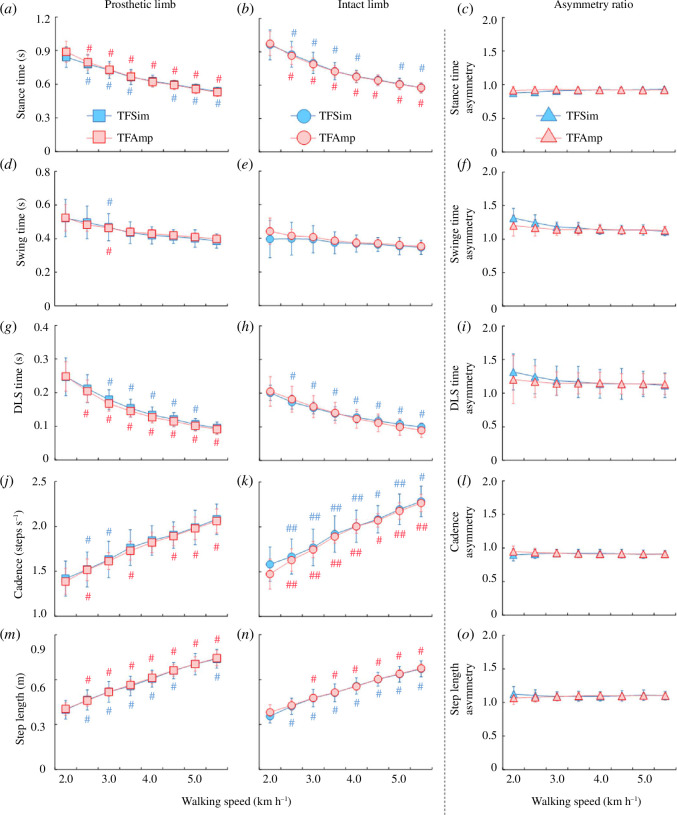
Comparisons of stance time (*a–c*), swing time (*d–f*), DLS time (*g*–*i*), cadence (*j–l*) and step length (*m–o*) for prosthetic (left) and intact (middle) limbs and their asymmetry ratio (right) between the TFSim and the TFAmp groups across a range of speeds. Blue and red indicate the TFSim and TFAmp groups, respectively. * and ** represent a significant difference between the TFSim and TFAmp groups at *p* < 0.05 and 0.01, respectively. (Note: They are not shown in this figure because no significant differences were found.) # and ## represent a significant difference between the current and previous speeds at *p* < 0.05 and 0.01, respectively.

**H1**: There was no statistical difference in stance time for prosthetic and intact limbs as well as in asymmetry ratios between the TFSim and TFAmp groups (figure 3*a*–*c*). **H2**: The main effect of speed on stance time was significant for prosthetic (χ^2^ (7) = 69.70, *p* < 0.001; χ^2^ (7) = 70.00, *p* < 0.001) and intact limbs (χ^2^ (7) = 69.70, *p* < 0.001; χ^2^ (7) = 70.00, *p* < 0.001) of both TFSim and TFAmp groups, respectively. In addition, there was a significant main effect of speed (*F*_(2.76,49.69)_ = 5.57, *p* = 0.003, ES = 0.24) and interaction (*F*_(2.76,49.69)_ = 4.32, *p* = 0.010, ES = 0.19) for the stance time asymmetry. However, no statistical differences in the asymmetry ratios were found between two consecutive speeds (electronic supplementary material, table S1).

#### 3.2.2. Swing time

**H1**: There was no statistical difference in swing time for prosthetic and intact limbs, as well as in asymmetry ratios between the TFSim and TFAmp groups (figure 3*d–f*). **H2**: There was a significant main effect of speed on swing time for prosthetic (χ^2^ (7) = 59.98, *p* < 0.001; χ^2^ (7) = 65.80, *p* < 0.001) and intact limbs (χ^2^ (7) = 50.50, *p* < 0.001; χ^2^ (7) = 52.37, *p* < 0.001) of both TFSim and TFAmp groups, respectively. In addition, the main effect of speed (*F*_(2.03,36.61)_ = 15.71, *p* < 0.001, ES = 0.47) and interaction (*F*_(2.03,36.61)_ = 4.05, *p* < 0.025, ES = 0.18) was found to be significant for swing time asymmetry. However, no statistical differences in the asymmetry ratios were found between two consecutive speeds.

#### 3.2.3. Double limb stance time

**H1**: There was no statistical difference in DLS time for prosthetic and intact limbs, as well as in asymmetry ratios between the TFSim and TFAmp groups (figure 3*g*–*i*). **H2**: There was a significant main effect of speed on DLS time for prosthetic (χ^2^ (7) = 69.63, *p* < 0.001; χ^2^ (7) = 70.00, *p* < 0.001) and intact limbs (χ^2^ (7) = 70.00, *p* < 0.001; χ^2^ (7) = 70.00, *p* < 0.001) of both TFSim and TFAmp groups, respectively. Although a significant main effect of speed (χ^2^ (7) = 49.28, *p* < 0.001) was found for DLS time asymmetry, there were no statistical differences between two consecutive speeds.

#### 3.2.4. Cadence

**H1**: There was no statistical difference in cadence for prosthetic and intact limbs, as well as in asymmetry ratios between the TFSim and TFAmp groups (figure 3*j*–*l*). **H2**: There was a significant main effect of speed on cadence for prosthetic limb (χ^2^ (7) = 67.73, *p* < 0.001; χ^2^ (7) = 69.63, *p* < 0.001) of both TFSim and TFAmp groups, respectively. On cadence for the intact limbs of both TFSim and TFAmp groups, there was a significant main effect of speed (*F*_(2.10,37.89)_ = 329.31, *p* < 0.001, ES = 0.95) but no interaction (*F*_(2.11,39.90)_ = 1.34, *p* = 0.28, ES = 0.07). There was no significant main effect of speed (*F*_(2.14,38.52)_ = 1.06, *p* = 0.36, ES = 0.06) or interaction (*F*_(2.14,38.52)_ = 2.93, *p* = 0.06, ES = 0.14) for cadence asymmetry. Consequently, no statistical differences in the asymmetry ratios were found between two consecutive speeds.

#### 3.2.5. Step length

**H1**: There was no statistical difference in step length for prosthetic and intact limbs, as well as in asymmetry ratios between the TFSim and TFAmp groups (figure 3*m*–*o*). **H2**: The main effect of speed on step length was significant for prosthetic (χ^2^ (7) = 69.70, *p* < 0.001; χ^2^ (7) = 70.00, *p* < 0.001) and intact limbs (χ^2^ (7) = 70.00, *p* < 0.001; χ^2^ (7) = 69.10, *p* < 0.001) of both TFSim and TFAmp groups, respectively. It was found that there were no main effects of speed (*F*_(2.16,38.83)_ = 0.74, *p* = 0.50, ES = 0.04) and interaction (*F*_(2.16,38.83)_ = 2.77, *p* = 0.07, ES = 0.13) for step length asymmetry. Consequently, there were no statistical differences in the asymmetry ratios between two consecutive speeds.

### 3.3. Ground reaction force components and asymmetry

#### 3.3.1. Mediolateral ground reaction force

**Figure 4.**
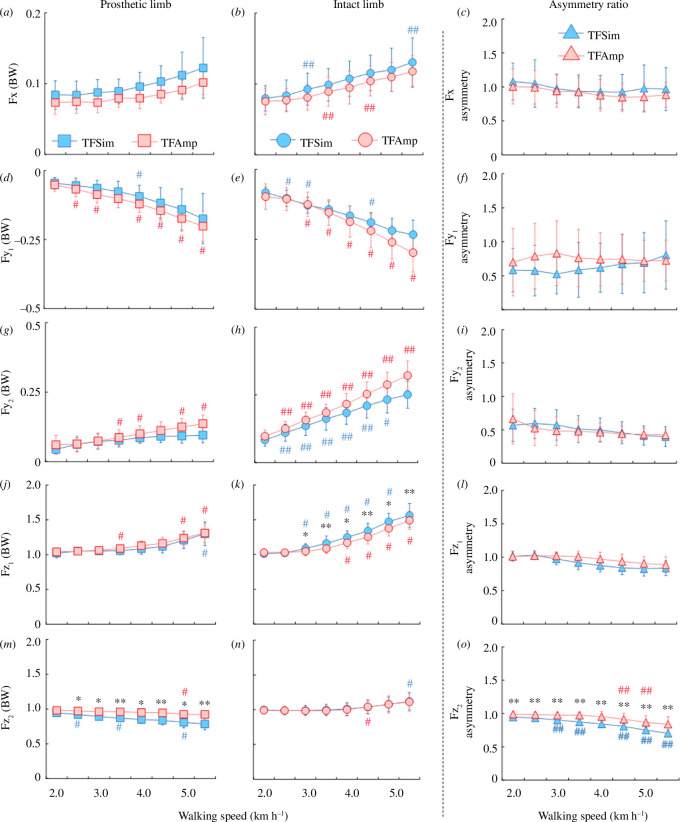
Comparisons of Fx (*a–c*), Fy1 (*d–f*), Fy2 (*g–i*), Fz1 (*j–l*) and Fz2 (*m–o*) for prosthetic (left) and intact (middle) limbs and their asymmetry ratio (right) between the TFSim and the TFAmp groups across a range of speeds. Blue and red indicate the TFSim and TFAmp groups, respectively. * and ** represent a significant difference between the TFSim and TFAmp groups at *p* < 0.05 and 0.01, respectively. # and ## represent a significant difference between the current and previous speeds at *p* < 0.05 and 0.01, respectively. BW, body weight.

**H1**: There was no statistical difference in mediolateral GRF (Fx) for prosthetic and intact limbs, as well as in asymmetry ratios between the TFSim and TFAmp groups (figure 4*a*–*c*). **H2**: A significant main effect of speed was found on Fx for the prosthetic limb (χ^2^ (7) = 34.30, *p* < 0.001; χ^2^ (7) = 69.63, *p* < 0.001) of both TFSim and TFAmp groups, respectively. On Fx for the intact limbs of both TFSim and TFAmp groups, there was a significant main effect of speed (*F*_(2.02,36.40)_ = 71.36, *p* < 0.001, ES = 0.80) but no interaction (*F*_(2.02,38.38)_ = 0.60, *p* = 0.56, ES = 0.03). Although a significant main effect of speed (χ^2^ (7) = 31.70, *p* < 0.001) was found for Fx asymmetry, there were no statistical differences between two consecutive speeds (electronic supplementary material, table S2).

#### 3.3.2. Anteroposterior ground reaction force

**H1**: There was no statistical difference in the first peak of anteroposterior GRF (Fy1) for prosthetic and intact limbs, as well as in asymmetry ratios between the TFSim and TFAmp groups (figure 4*d*–*f*). **H2**: A significant main effect of speed was found on Fy1 for prosthetic (χ^2^ (7) = 63.88, *p* < 0.001; χ^2^ (7) = 69.93, *p* < 0.001) and intact limbs (χ^2^ (7) = 68.47, *p* < 0.001; χ^2^ (7) = 68.90, *p* < 0.001) of both TFSim and TFAmp groups, respectively. There were no significant main effects of speed (*F*_(1.98,35.67)_ = 1.17, *p* = 0.32, ES = 0.06) and interaction (*F*_(1.98,35.67)_ = 3.24, *p* = 0.05, ES = 0.15) for Fy1 asymmetry. Consequently, no statistical differences in asymmetry ratios were found between two consecutive speeds.

**H1**: Although a significant main effect of group (*F*_(1,18)_ = 4.77, *p* = 0.042, ES = 0.21) and interaction (*F*_(1.63,30.97)_ = 5.74, *p* = 0.011, ES = 0.24) on the second peak of anteroposterior GRF (Fy2) was found for prosthetic and intact limbs, there were no statistical differences in Fy2 between the TFSim and TFAmp groups (figure 4*g,h*). Consequently, there were no statistical differences in Fy2 asymmetry ratios between TFSim and TFAmp groups (figure 4*i*). **H2**: There was a significant main effect of speed on Fy2 for prosthetic limb (χ^2^ (7) = 48.93, *p* < 0.001; χ^2^ (7) = 65.67, *p* < 0.001) of both TFSim and TFAmp groups, respectively. The Fy2 also showed a significant main effect of speed (*F*_(1.63,29.34)_ = 261.22, *p* < 0.001, ES = 0.94) for the intact limbs of both TFSim and TFAmp groups. A significant main effect of speed (*F*_(1.79,32.14)_ = 8.38, *p* = 0.002, ES = 0.32) but not interaction (*F*_(1.79,32.14)_ = 1.46, *p* = 0.246, ES = 0.08) was found for Fy2 asymmetry. However, there were no statistical differences in the asymmetry ratios between two consecutive speeds.

#### 3.3.3. Vertical ground reaction force

**H1**: There was no statistical difference in the first peak of the vertical GRF (Fz1) for prosthetic limb as well as in asymmetry ratios between the TFSim and TFAmp groups (figure 4*j,l*). However, there was a statistical difference in Fz1 for the intact limb between the groups at 3.0 km h^−1^ or higher speeds (figure 4*k*). **H2**: There was a significant main effect of speed on Fz1 for prosthetic (χ^2^ (7) = 50.27, *p* < 0.001; χ^2^ (7) = 60.77, *p* < 0.001) and intact limbs (χ^2^ (7) = 69.00, *p* < 0.001; χ^2^ (7) = 63.65, *p* < 0.001) of both TFSim and TFAmp groups, respectively. There were significant main effects of speed (*F*_(2.06,37.16)_ = 32.03, *p* < 0.001, ES = 0.64), but no interaction (*F*_(2.06,37.16)_ = 3.22, *p* = 0.51, ES = 0.15) for Fz1 asymmetry. However, no statistical differences were found in the asymmetry ratios between two consecutive speeds.

**H1**: There were statistical differences in the second peak of vertical GRF (Fz2) for the prosthetic limb between the TFSim and TFAmp groups at all speeds (figure 4*m*). However, there was no statistical difference in Fz2 for the intact limb between the groups (figure 4*n*). There was a significant main effect of group (*F*_(1,18)_ = 17.31, *p* < 0.001, ES = 0.49) and interaction (*F*_(1.61,29.08)_ = 4.356, *p* = 0.029, ES = 0.20) for Fz2 asymmetry. Consequently, there were statistical differences in asymmetry ratios between the groups (figure 4*o*). **H2**: There was a significant main effect of speed on Fz2 for the prosthetic limb (χ^2^ (7) = 67.87, *p* < 0.001; χ^2^ (7) = 47.67, *p* < 0.001) of both TFSim and TFAmp groups, respectively. There was a significant main effect of speed (*F*_(1.53,27.52)_ = 20.71, *p* < 0.001, ES = 0.54) but no interaction (*F*_(1.53,29.07)_ = 0.06, *p* = 0.91, ES = 0.01) on Fz2 for the intact limbs of both TFSim and TFAmp groups. In addition, there was a significant main effect of speed (*F*_(1.61,29.08)_ = 81.35, *p* < 0.001, ES = 0.82) for Fz2 asymmetry. The Fz2 asymmetry was statistically increased at all speeds except 2.5 and 4.0 km h^−1^ in the TFSim, and only at 4.5 and 5.0 km h^−1^ in the TFAmp group, when compared with the speed of the previous stage.

## 4. Discussion

This study aimed to investigate the differences in gait parameters and asymmetry ratios between TFSim and TFAmp groups at each individual speed, as well as the effects of gradual changes in gait speed on these parameters when comparing the two groups. Our first hypothesis was that there would be significant differences in gait parameters and asymmetry ratios at each individual speed. As shown in figures 3 and 4, there were no differences in most of the spatio-temporal and GRF parameters except Fz1 for the intact limb and Fz2 for the prosthetic limb between the TFSim and TFAmp groups. Additionally, there were no differences in the asymmetry ratios of those parameters except Fz2 between the groups. These findings are also in agreement with a previous study on a list of biomechanical parameters (i.e. joint angular velocities, moments and powers), which found similarities between the TFSim and TFAmp groups [8]. Therefore, the vertical GRF parameters collected using the TFSim should be interpreted with caution. Our second hypothesis was that an increment in gait speed would have significantly different effects on gait parameters and asymmetry ratios when comparing the two groups. There were significant main effects and subsequent differences between two consecutive speeds within each group on most spatio-temporal and GRF parameters and their asymmetry ratios from slow to fast walking. These outcomes suggested that walking speeds would have similar impacts on gait parameters and asymmetry in the TFSim and TFAmp groups.

In agreement with the previous studies on individuals with unilateral TFAmp, this study demonstrated a longer stance and shorter swing time in the intact limb compared with the prosthetic limb (figure 3*a,b,d,e*) [25,35,36]. The stance and swing time asymmetry ratios for both the TFSim and TFAmp groups were close to 1.0 when walking at 3.0 km h^−1^ or higher, with no significant differences between the two groups (figure 3*c,f*). The gait asymmetry ratios displayed for the TFAmp group over a range of walking speeds were consistent with a previous study [25]. There were also no significant differences in DLS time and its asymmetry ratios across speeds between the TFSim and TFAmp groups. A previous study also reported a similar asymmetrical trend in individuals with unilateral TFAmp [37].

No differences in step length and its asymmetry were found between the two groups (figure 3*m*–*o*). A previous study on individuals wearing TFSim also reported no significant differences in step length when compared with individuals with unilateral TFAmp [8]. Additionally, there were no differences in step length and cadence for prosthetic and intact limbs between the TFSim and TFAmp groups and their subsequent changes with speeds (figure 3*j,k,m,n*), which are consistent with the results of the previous studies [21,38,39]. It appears that the step length and cadence and their asymmetry ratios are similar between the TFSim and TFAmp groups across a range of walking speeds. Thus, the TFSim may be capable of simulating the spatio-temporal parameters of gait in individuals with unilateral TFAmp.

The changes in Fx, Fy1 and Fy2 for prosthetic and intact limbs as well as in their asymmetry ratios between TFSim and TFAmp groups might be supported by previous studies that reported similar changes across walking speeds [25,40]. Similar asymmetry patterns of mediolateral GRF in the TFSim and TFAmp groups were revealed in a study that compared individuals with unilateral TFAmp and non-disabled individuals [25]. However, the irregular effects of speed within each group may be related to changes in muscular and proprioceptive control, regulating the whole-body centre of mass acceleration and GRF generation [22,41]. Although there was no significant difference in the asymmetry ratio, the Fz1 in the intact limb was larger than that in the prosthetic limb in the TFSim group at faster walking speeds (figure 4*j,k*). This finding was supported by a previous study that reported that vertical GRF during the weight acceptance phase was significantly larger in non-disabled individuals than in individuals with unilateral TFAmp [42]. Similarly, across all tested walking speeds, Fz2 for the prosthetic limb as well as its asymmetry ratios were also significantly different between the TFSim and TFAmp groups. This might be owing to the mechanism of prosthetic knee joints and the structural asymmetries between the prosthetic limbs of the TFSim and TFAmp groups. The differences in Fz1 and Fz2 magnitude as well as in their asymmetry ratios between TFSim and TFAmp groups for the intact limb and prosthetic limb, respectively, might be attributed to these structural and functional asymmetries (figure 4*k,m*,*o*). It is noteworthy that the absence of differences in the asymmetry ratio of Fx, Fy1 and Fy2 between TFSim and TFAmp is probably attributed to their wider distributions. The vertical component of GRF usually constitutes a larger proportion, making it more stable during walking, while the forces in the anteroposterior and mediolateral directions have smaller components, making them more susceptible to inconsistencies and disturbances. Therefore, the TFSim may not be able to realistically reproduce the gait of an individual with unilateral TFAmp during weight acceptance and push-off phases of walking particularly at faster speeds. However, given the potential limitations of the study hypotheses, these results should be approached with caution and further validated in future research.

The prosthetic knee joints used in this study (3R95, 3R80, 3R60, 3R106, Swan100, Dolphin and Total Knee) typically allow flexion of the joint during the swing phase while stabilizing the joint during the stance phase [43]. The type of prosthetic knee and foot joints might impact the anteroposterior force, and the way the knee is flexed at late stance may also affect the anteroposterior force [44]. However, the existing evidence supporting this assertion is currently limited and requires further studies to explore the impacts of the various types of knee joints on gait kinetics, kinematics and spatio-temporal parameters. The prosthetic limb of non-disabled individuals consists of a TFSim and their entire lower limb, whereas the prosthetic limb of individuals with unilateral TFAmp consists of only a transfemoral prosthesis and the residual limb. The mass of the shank and foot segments of non-disabled individuals is estimated to be 6.1% of the total body mass [45]. The discrepancy in mass between the TFSim and TFAmp groups could be further accentuated by the shortening of the amputee’s residual limb. Additionally, the unique socket designs in TFSim to accommodate the entire lower limb could further affect the mass of the prosthetic limb in TFSim groups. These could introduce a larger value of the vertical GRF loading on the prosthetic limb in the TFSim group when compared with the TFAmp group. Therefore, adapting limbs to a TFSim could alter the thigh segment’s centre of mass position and inertia. This could result in larger variations in local joint dynamics when compared with individuals with TFAmp [28,46,47]. The individuals with TFSim may need to apply additional force during push-off with the prosthetic limb and subsequent weight acceptance with the intact limb to flex the prosthetic knee joint to transit into the swing phase. The individuals with unilateral TFAmp, in contrast, require force to transit the prosthesis-only limb (with their residual limb) into the swing phase.

Furthermore, in the intact limb, the vertical GRF of individuals with unilateral TFAmp was reported to be smaller than that in non-disabled individuals [25,48]. Non-disabled individuals wearing a TFSim with the reduced degrees of freedom of the prosthetic segment attempt to modulate their intact limb rather than the prosthetic limb to adjust their gait pattern [39]. Adaptive strategies for modulating the intact limb when flexing the prosthetic knee joint to clear the ground and then extending for heel strike may result in different vertical GRF patterns. The larger spinal loads during push-off with the intact limb in individuals with TFAmp emphasizes a complex pattern of trunk muscle recruitment and could pose a greater risk of getting fatigued from frequent exposure to the additional loading [42,49]. Thus, repeated walking at faster speeds could lead to fatigue in the intact limb and might influence the vertical GRFs in individuals with unilateral TFAmp. Other factors that could create significant differences in vertical GRFs may be the variation in the structural symmetry of prosthetic limbs, forces applied and physical differences in intact limbs between non-disabled and amputated individuals. Therefore, the use of TFSims may warrant a more rigorous investigation into the causes of variations to effectively simulate the walking of individuals with unilateral TFAmp in terms of GRFs, particularly vertical components.

Even though the TFSim and TFAmp groups showed some notable similarities in spatio-temporal and part of GRF parameters as well as their asymmetry ratios, this may not be enough to support a reliable model of prosthetic gait. The TFSim’s interaction with non-disabled individuals differs from that of individuals with unilateral TFAmp in terms of the location of the effective knee joint, limb/socket interface, etc., that may affect walking mechanics [27,28]. Individuals with unilateral TFAmp have a critical compensation gait strategy that decreases the hip range of motion on the prosthetic side and increases anterior pelvic tilt on the intact side [50]. These changes alter the local joint dynamics that differ from the amputated thigh, regardless of whether the asymmetrical gait measurements are similar between the TFSim and TFAmp groups or not. For instance, when non-disabled individuals using transtibial prosthetic simulators were compared with individuals with unilateral transtibial amputation in similar research settings, contentious findings were reported [51,52]. Using the same transtibial prosthetic emulators but different prosthetic sockets, researchers observed that as push-off increased, metabolic expenditure increased in non-disabled participants but not in individuals with unilateral transtibial amputations. Therefore, for TFSims to be a realistic means to study how novel prosthetic componentry affects spatio-temporal parameters and symmetry in TFAmp, other gait parameters may also need to be compared with check the similarity between the TFSim and TFAmp groups [53]. Nevertheless, TFSims could serve as a convenient tool for prototyping novel prosthetic components in the initial stage of development before recruiting individuals with unilateral TFAmp.

There were some limitations in this study. The participants in this study were notably young and highly active, which could potentially skew the parameters analysed positively. The sample size for the study was relatively small and was not chosen based on statistical prior analysis. The participants affiliated with a certain institution may have exhibited bias. This study neither confirmed the effect of controlled changes on prosthetic interventions nor matched them between the TFSim and TFAmp groups. The use of an instrumented treadmill instead of authentic overground walking might also have influenced corresponding spatio-temporal and GRF parameters. Additionally, employing significance tests to establish equivalence may present challenges. The abundance of comparisons could weaken the robustness of the findings. Considering the hypotheses that there are differences between the interventions, instances where no significant difference is observed in most comparisons might indicate a lack of support for the hypotheses. Both L-shaped and V-shaped TFSims were used; however, the effects of the shape on gait are unknown. In addition, the current study analysed only the spatio-temporal and GRF parameters of gait. Future research should investigate gait in terms of kinematics and muscle activity, as well as other kinetic parameters such as joint torque and/or mechanical work that are also critical in the gait rehabilitation of individuals with amputation. Therefore, future studies should be conducted with a larger sample/age size and a wide range of gait parameters to comprehensively investigate the differences between individuals with unilateral TFAmp and individuals wearing a TFSim. In future research, investigating the training process of non-disabled individuals using TFSim to attain a stable gait pattern could aid in assessing the required training duration for appropriate inclusion criteria in similar studies.

## 5. Conclusion

This study found no significant differences in gait parameters such as spatio-temporal, mediolateral GRF and anteroposterior GRF when comparing individuals wearing a TFSim and individuals with unilateral TFAmp using a prosthesis. However, a significant difference was found in the first (Fz1: intact limb) and second (Fz2: prosthetic limb) peaks of the vertical GRF as well as in the Fz2 asymmetry ratio during push-off. This may be owing to the function of prosthetic knee joints, variation in the settings of prosthetic limbs, the difference in movements of the intact limb, strength and level of fatigue between the TFSim and TFAmp groups. It could be concluded that the use of a TFSim might be effective in reproducing the gait pattern of an individual with unilateral TFAmp regarding temporal parameters such as stance time, swing time, DLS time and cadence; spatial parameters such as step length; and GRF parameters such as mediolateral and anteroposterior GRFs. Caution should be exercised when assessing the asymmetry ratio of Fx, Fy1 and Fy2. However, the vertical GRFs at weight acceptance and push-off phases of walking with a TFSim might not be reproduced to match those in individuals with unilateral TFAmp using a prosthesis. Therefore, vertical GRF parameters warrant further study to reveal the underlying mechanism of effectively simulating the walking of individuals with unilateral TFAmp via using a TFSim.

## Data Availability

The electronic supplementary materials for this article, including individual data, figures, tables, and movies are provided as follows: electronic supplementary material, data S1. Individual data of all parameters at each walking speed; electronic supplementary material, figure S1. Individual comparisons of GRFs between the TFSim and TFAmp groups based on the type of knee joint; electronic supplementary material, table S1. Main effects and interaction for the spatio-temporal parameters and asymmetry; electronic supplementary material, table S2. Main effects and interaction for the GRF parameters and asymmetry; electronic supplementary material, video S1. How to don a TFSim; electronic supplementary material, video S2. Ten individuals with TFSim walking at 3.5 km h^−1^ on the treadmill; and electronic supplementary material, video S3. Ten individuals with TFAmp walking at 3.5 km h^−1^ on the treadmill [54].
